# The rediscovery of Fritz Heider’s early social cognition: a person-centered perspective

**DOI:** 10.3389/fpsyg.2025.1577720

**Published:** 2025-05-21

**Authors:** Yaming Shang, Da Dong, Wei Chen

**Affiliations:** ^1^Department of Mathematics, Shaoxing University, Shaoxing, China; ^2^Center for Brain, Mind and Education, Shaoxing University, Shaoxing, China; ^3^Department of Psychology, Shaoxing University, Shaoxing, China

**Keywords:** animacy perception, Fritz Heider, common-sense psychology, cognitive revolution, person perception, social perception, agency

## Abstract

In this article, we situate the social psychologist and philosopher Fritz Heider’s theory within what we call “early social cognition,” a historical approach preceding and radically differing from contemporary “social cognition.” By incorporating recent developments in issues such as perception, animacy, and social structures (networks), we reassess key aspects of Heider’s system to demonstrate their present-day significance. This analysis does not merely reiterate Heider’s ideas but shifts the methodological focus from his causal analysis of event attribution to a constitutive scientific explanation. In particular, we examine Heider’s early focus on the perceptual realism of the general object and his emphasis on the person-thing distinction. By engaging with contemporary developments on the animate-inanimate subcategorical distinction, we argue that Heider’s person-centered perspective may offer a unified theoretical framework for the construction of theories in social cognition.

## 1 Introduction

…social cognitive psychology will never be a literal translation of cognitive psychology.

Fiske and Taylor (1984, p. 17)

Viewed through the broader historical lens of scientific paradigm shifts, contemporary debates in social cognition reflect a continuation of the enduring tension between the first-generation cognitive science (or the standard cognitive science) and the second-generation cognitive science (Gallagher, 2020; Marr, 1982; Shapiro, 2011; Varela et al., 1991). Social cognition, having adopted the fundamental tenets of cognitivism (i.e., information processing psychology), also inherits its entrenched theoretical burdens. Since the 21st century, the rebellion of new theories (including direct social perception, interactionism, and social enactivism) has emerged as a response to this enduring historical impasse (Chen et al., 2024; Schlicht, 2023; Zahavi, 2014, 2023). When theories of “social cognition” are examined within the internal history of social psychology, their conceptual underpinnings may call for critical reinterpretation through this internalist perspective. While such an approach necessarily rejects a Whiggish narrative of scientific progress, it also underscores the significance of one academic community’s contributions during a particular historical moment.

A commonly overlooked historical fact, particularly by philosophers of cognitive science after the 1980s, is that well before the cognitive revolution of 1956—even during the predominance of behaviorism and neo-behaviorism—many social psychologists were already addressing social psychological questions through a distinctly cognitive framework. Their work moved beyond mere rhetorical gestures, advancing toward an independent and systematic mode of thought. Traces of this history can still be found in the recollections of pioneers of the cognitive revolution, such as George Miller and Jerome Bruner. In his reflections, Miller not only highlighted the dominance of behaviorism within experimental psychology but also emphasized that social and clinical psychology were not within behaviorism’s scope of domination (Baars, 1986, p. 203). Similarly, Bruner (1983), recalled participating in a 1955 symposium titled “Contemporary Approaches to Cognition.” He regarded this symposium as a typical example of the cognitive trends of the 1950s. However, in comparison to the famous Dartmouth Workshop on “Artificial Intelligence” held a year later, widely recognized as the starting point of the 1956 cognitive revolution, Bruner considered his presentation at the 1955 symposium as merely “an early and rather half-baked version of the style of thinking of the ‘cognitive revolution’” (p. 115). The papers presented at the 1955 symposium were subsequently compiled into a collection under the same title and published in 1957. In the concluding remarks of this collection, Fritz Heider, a central figure at the symposium and during this early social cognition period, proposed that this collection might represent the first compilation focusing on “cognition”: “recently, interest has been revived in problems related to cognition. One sign is this symposium on cognition; I think somebody said that this is the first symposium on cognition.” (Bruner et al., 1957, p. 203).

In contemporary discourse, several seminal textbooks on “social cognition” or “social psychology” continue to highlight the historical shift during the 1950s, when a significant number of social psychologists departed from the tenets of behaviorism. For instance, Fiske and Taylor (1984) noted, “experimental psychology rejected cognition for many years, while social psychology did not” (p. 6), as well as “in contrast to experimental psychology, social psychology has consistently leaned on cognitive concepts, even when most psychology was behaviorist” (p. 8). Kunda (1999) observed that, although behaviorism dominated experimental psychology at the time, social psychologists were indifferent to behaviorist views, and “cognition” remained central to social psychology’s theories and research (p. 2). Similarly, Hogg and Vaughan (2018) claimed, “… in many ways, social psychology has always been fundamentally cognitive in its perspective” (p. 22).

In this article, we situate Heider’s theory within what we call “early social cognition,” a historical approach preceding and radically differing from contemporary “social cognition.” By incorporating recent developments in issues such as perception, animacy, and social structures (networks), we reassess key aspects of Heider’s system to demonstrate their present-day significance. This analysis does not merely reiterate Heider’s ideas but shifts the methodological focus from his causal analysis of event attribution to a constitutive explanation. Our approach emphasizes the subcategorical distinction between “person” and “thing” as intrinsic properties of objects. Here, “person” refers broadly to any entity capable of autonomous action and social interaction, not limited to human individuals. Conversely, “thing” is defined in opposition to this concept of “person” (see Fiske and Taylor, 1984; Heider, 1958). By engaging with contemporary developments on the animate-inanimate subcategorical distinction, we argue that Heider’s person-centered theory may offer a unified theoretical framework for the construction of theories in social cognition.

## 2 A person-centered early social cognition

The leading figures of early social cognition, active from the 1940s to the early 1950s, included Solomon Asch, Bruner, Egon Brunswik, Leon Festinger, Heider, Kurt Lewin, Martin Scheerer, etc. Many of them, such as Asch, Brunswik, Festinger, Heider, and Lewin, either belonged to or maintained close ties with the Gestalt school (Hilton, 2012; Patnoe, 1988; Sahakian, 1982).

In early social cognition, “cognition” was not a strictly defined concept; scholars encouraged more open and diverse ideas to foster the development of new approaches. Scheerer (1954) defined “cognition” as: “cognitive theory might be expected to deal with the problem of how man gains information and understanding of the world about him, and how acts in and upon his environment on the basis of such cognitions” (p. 91). Festinger (1957) defined “cognition” as: “by the term *cognition*…I mean any knowledge, opinion, or belief about the environment, about oneself, or about one’s behavior” (p. 3). Heider (1958) explicitly used the term “cognitive psychology,” stating: “the idea that our cognitions, expectations, and actions are based on a mastery of the causal network of the environment, is, of course, the main tenet of a cognitive psychology…” (p. 59) Heider (1957) identified four points of consensus on “cognitive psychology” that emerged from the heated discussions among the contributors: (1) despite differences in terminology, authors shared a focus on the relationships between variables occurring in the input–output process; (2) symbolic representation (although in a very primitive sense); (3) the relationship between outputs and cognitive processes or representations. Heider emphasized that proponents of this new approach needed to pay greater attention to the empirical grounding of their theories; (4) the underlying structure of cognition. Terms such as “codes” and “coding systems” were frequently used. Regarding the future of “cognitive psychology,” Heider expressed optimism about the future of “cognitive psychology,” proposing that the lack of consensus in the symposium was advantageous, as it suggested a dynamic process of intellectual progress with developments trending in a promising direction (pp. 209–210).

In retrospect, from the vantage point of subsequent, matured information processing psychology, this compendium of papers, while encompassing numerous theoretical components of significance, may appear somewhat rudimentary (as Bruner noted). The participants, drawing on their respective previous knowledge and experiences, began to familiarize themselves with new concepts such as “coding” (computation), “representation,” and “information.” In some sense, this approach fell short of the thoroughness characteristic of the representationalism-computationalism approach that would become the standard of cognitive science in later years. In summary, from the Whiggish historical perspective of the standard cognitive science, the 1955 symposium appears to have been an internal rehearsal within the field of psychology—one that was ultimately unsuccessful—preceding the landmark 1956 summer workshop.

In early social cognition, the concept of “cognition” fundamentally served to understand what the “person” is, rather than aiming to establish a general abstraction principle of cognition based on the computer metaphor of information processing divorced from the actual entity “person.” Heider (1957) remarked that his common-sense psychology was intended to lay the groundwork for scientific (social) psychology. He criticized Brunswik, a highly influential theoretical psychologist of this period who was deeply influenced by Heider’s theory of perception, arguing that “he restricted himself more or less to perception and, one might add, to the perception of objects” (p. 201). Moreover, Heider expressed some reservations about the broad influence of the concept of “information,” noting that “now it is still not clear how much information theory has to do with cognitive processes, since it does not concern itself with meanings” (p. 204). In early social cognition, the investigation of what a “person” is and how a “person” can be perceived was of primary importance. As Miller observed, early social cognitivists such as Heider and Festinger “were still interested in people, and would stoop to any way to learn more about them” (Baars, 1986, p. 212), prioritizing the study of the person over strict adherence to any particular theory, model, or metaphor.

The 1955 symposium failed to produce a unified definition of “cognition,” leaving participants to pursue divergent lines of inquiry (Tagiuri and Petrullo, 1958). While figures like Bruner did not pursue the path endorsed by Heider, this diversity was precisely the condition Heider believed necessary for fostering a dynamic and progressive research environment.

## 3 Theoretical components of Heider’s system: a re-formulation

In the early 1980s, during the golden age of information processing psychology’s dominance over social psychology, social psychologists discussing the prospects of social cognition, such as Ostrom (1984), were highly optimistic about overcoming the theory crisis and regaining autonomy by adopting the cognitivist tenets. At that time, the limitations of information processing psychology had not yet reached a consensus within the community. However, the historical contributions and significance of early social cognition had already been deliberately downplayed (though not entirely forgotten). The history and theory of early social cognition were either deliberately ignored by social psychologists of the 1980s or overlooked out of ignorance by some philosophers of cognitive science. This has led to its marginalization and the subsequent influence on the reconstitution of the new classical philosophical frameworks of social cognition, particularly theory theory and simulation theory. Subsequently, contemporary social cognition faced a theoretical challenge: the general abstraction principle of cognition derived from information processing psychology could not be seamlessly applied to social cognition. To put it plainly, the “thing”-centered information processing failed to adequately address the demanding need of social cognition—“would stoop to any way to learn more about” persons—and offered little assistance in this regard.

In Heider’s early work on social cognition, he posited a fundamental assumption: the “person” as an object of perception and the “thing” as an object of perception—while both falling under the category of general object—are subcategorically distinct. This thesis, therefore, involves two key theoretical components in its delineation of perceptual objects: the definition of “general object” and the distinction between person and thing as objects (we will argue the latter corresponds to the animate-inanimate distinction in contemporary discourse). Among early scholars of social cognition, Heider’s framework arguably provides the most reliable starting point for two main reasons: first, it integrates both the perception of general objects and the perception of persons, offering a more comprehensive account (whereas others typically addressed only one of these aspects); second, his general object theory is the earliest foundational contribution within early social cognition, exerting significant influence on later prominent theories. Heider’s early work emphasized the distinction between general perceptual objects and mediums (Heider, 1920, 1926), while his later work systematically developed accounts of person perception and what he referred to as common-sense psychology (Heider, 1958; Heider and Simmel, 1944). The two subsequent major theories of objects—Brunswik’s probabilistic functionalism and James Gibson’s ecological psychology—can both be regarded, to some extent, as derivative extensions influenced by Heider’s initial theory (Heft, 2001; Wieser, 2014).

In Table 1 we provide a summary of Heider’s person-centered system in early social cognition. It is worth emphasizing that, although Heider (1958) presupposed a distinction between the person and the thing based on their properties of animacy, this distinction was not thoroughly analyzed at the time. Contemporary advances in neurotechnology now allow the inclusion of hierarchies of animacy as an analysis of objects *per se*.

**Table 1:** Components of Heider’s person-centered system.

Object	Components of theory	Domain
General object	Causal analysis of perception	Perceptual realism
Person-thing distinction	Hierarchies of animacy	Cognitive neuroscience
Event	Attribution	Common-sense or naive psychology
Person–person relations, or person-society relations	Social units analysis	Scientific social psychology

Table 1 is taken from Dong and Chen (2025, p. 178).

### 3.1 General objects

Briefly, Heider’s writings, such as Heider (1920) and the later revised versions in Heider (1926, 1959), represent a systematic response to his mentor Alexius Meinong’s (1906) problem of perceptual realism. This issue inaugurated the pathway for the causal analysis of perception: under conditions of adequate lighting and a clear visual field, the visual stimuli received by our sensory surfaces can be traced almost entirely to photons within the light array. Why, then, do we not refer to the final source of the visual stimuli and claim to perceive light *per se* (or simply, the sun)? Instead, we assert that we perceive the diverse objects on Earth—objects that result from light being reflected at intermediate stages. Put simply, what kind of “object” is causally responsible for perceptual or cognitive processes? (Heider, 1983, p. 36). Here, the object is understood as the result of causal analysis in perceptual attribution. Heider distinguished between object and medium. The light array constitutes the medium between the organism and the environment. Adopting a realist stance, Heider proposed the concept of the “spurious unit.” He argued that we refer to what we perceive as objects, rather than the non-object—medium, because the medium constitutes “spurious unit” that cannot be perceived as wholes (Heider, 1959). In other words, perceptual attribution ultimately depends on the discernible intrinsic structures of objects *per se*.

While the general object theory and perceptual causal analysis are not the primary focus of this paper, they provide a foundational premise for Heider’s person-centered social cognition. The distinction between “person” and “thing” as perceived objects, despite their evident differences in constitutive properties, situates both within the broader framework of general object theory as distinct subcategories. As Heider noted:

“we shall speak of ‘thing perception’ or ‘nonsocial perception’ when we mean the perception of inanimate objects, and of ‘person perception’ or ‘social perception’ when we mean the perception of another person. …the word ‘object’ is also used in its more general sense—‘the object of perception’ or ‘the distal object’—which includes persons as well as things.” (Heider, 1958, p. 21)

Thus, before analyzing the differences between the social “person” and the nonsocial “thing,” it is crucial to first recognize their common characteristics.

### 3.2 Event attribution

Heider (1958) drew a clear distinction between his focus on person perception and the traditional themes of social psychology. He argued that social psychology had previously addressed superindividual entities, social polyad, or the relationship between persons and social unity. In contrast, his investigation centered on one person’s perception of another person, even outside the constraint of a dyad, as illustrated by his claim: “the discussion will center on the person as the basic unit to be investigated. …the two-person group and its properties as a superindividual unit will not be the focus of attention” (p. 1).

While adopting a realist position to distinguish between object and medium, Heider maintained that medium cannot be perceived because it fails to form a unit of perception corresponding to its realist nature. Yet, in the domain of social cognition, he distinguished person perception from thing perception through the method of event attribution. The distinction between social and nonsocial events lies in the fact that the former includes a “person” as the most important constitute for explanation. This analytical method is indirect, as it does not directly address the distinctions in the properties of the perceived “person” or “thing” as objects. In event attribution, the “person” is treated as a holistic unit applied to events without any analysis of its constituents. Early in his investigation, Heider (1958) presupposed differences in animacy between these two types of objects but did not further explore their distinctions concerning hierarchies or continuity. For example, Heider (1958, pp. 107−108) analyzed event causality involving these two distinct types of objects. One analysis concerns event (*x*) that does not involve a “person,” where different circumstances (e.g., *c1*, *c2*, *c3*) produce different effects (e.g., *e1*, *e2*, *e3*). Here, a given circumstance causally produces an effect in a causally forward manner, marked as: *e* → *c*. Another analysis illustrates human goal-directed behaviors. In this case, *x* indicates an event involving human action. Even under varying circumstances, the same person may employ different means (*m*) to achieve the same effect or goal (*e*). In this sense, the causal distinction in human perception lies in the fact that rather than circumstances causing effects, an effect (goal *e*) exerts a causally backward influence on the selection of means (*m*) in given circumstances (*c*). This can be represented as: *e* → *m*/*c*.

Event attribution forms the main content of Heider’s common-sense psychology, which is intended to lay the groundwork for scientific social psychology. His work inaugurated the study of attribution within the history of social psychology. However, as emphasized in the preceding discussion, from the perspective of the constitutive explanation concerning the general object and the person-thing distinction, event attribution merely situates a person or thing as a whole within an event as the explanatory constituent of the attribution, without analyzing the more fundamental causes of the expressive/perceptual attributes of the person itself.

This explanatory gap, based on Heider’s texts, primarily lies in animacy perception. Before perceiving a specific mental state or content, we must first perceive this entity as mental or minded (as opposed to physical) (Varga, 2017, 2020); just as, before perceiving a person’s specific mental states and interacting with this person, we must first identify him/her as a “person” (rather than a thing). However, the “identification” in question here does not pertain to high-level cognitive processing involving representations or propositional attitudes but rather involves making a basic distinction between the animate and the inanimate, based on simple cues (e.g., shape or kinematics) (Parovel, 2023).

### 3.3 Hierarchies of animacy

The distinction between physical causality and purposiveness in social events and nonsocial events involving two different objects cannot replace a constitutive explanation of the objects *per se*. Analyzing and generalizing the intentions behind an agent’s actions toward another agent is not, strictly speaking, a matter of person perception, but relates more to social contexts, personal beliefs, attitudes, and experience-based knowledge. A “person” is not a “thing”; and these two types of objects must belong to a unified conceptual continuum, within which they are categorized under separate subdomains. In contemporary discourse, this overarching conceptual framework is termed “animacy.” As Heider (1958) demonstrated in distinguishing between “person” and “thing,” this framework functions as a presupposition (p. 21). However, the dichotomy between perceiving a “person” and perceiving a “thing”—as the perceptual foundation for social cognition—is not self-evident. Put simply, before perceiving a person’s mental states, one must first identify it as a “person” rather than as a “thing.” Absent such recognition, social cognition about a “person” would be impossible.

In psychology, the perception of objects involves phenomenal causality and animacy (Heider and Simmel, 1944; Michotte, 1946/1963), which have become distinct and independent fields of inquiry. The work of Heider and Simmel (1944) is regarded as the starting point of modern animacy research (Rutherford and Kuhlmeier, 2013; Scholl and Tremoulet, 2000). Their experiments implied that subjects almost invariably anthropomorphized the interactions of several geometric shapes in the animation as interpersonal relationships, perceiving these shapes as being “alive.” Heider and Simmel’s experiments utilized simple kinematic cues from two-dimensional figures to elicit perceptions of animacy. Moreover, geometric shapes *per se* can function as primitive types of percepts of social cognition, as seen in human face recognition or emoji use in online communication.

**Figure 1:**
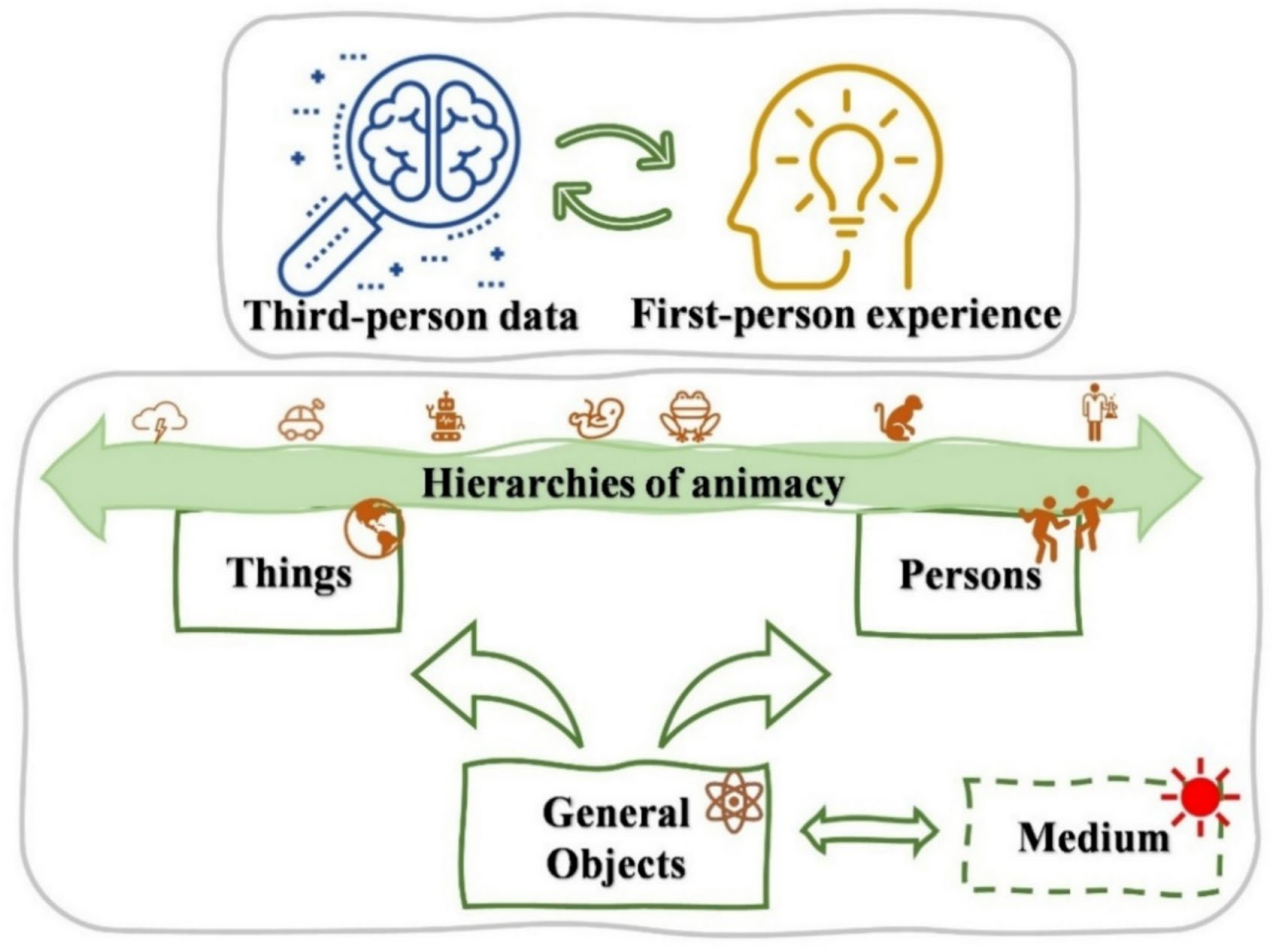
The theory part which reformulates the distinction between “person” and “thing” objects in Heider’s system. Subjects can perceive first-person experiences related to animacy, while neural events occurring in the brain can be captured as third-person data through neurotechnology. This approach parallels the neurophenomenological method employed in consciousness studies.

To date, the concept of animacy remains without a universally accepted, precise definition. In contemporary use, animacy generally refers to the capacity to perceive an object as a “living” agent. However, the terms “living” and “agent” are poorly defined as well. Currently, three main approaches to defining animacy exist. (1) The traditional biotic approach equates the animate-inanimate distinction in common-sense psychology with the living-nonliving distinction in naive or scientific biology (Westfall, 2023). Yet, the concept of “living” is vague and ambiguous across linguistic and cultural contexts. As early as the 1940s, Chinese psychologist I. Huang criticized Jean Piaget’s theory of child animism—which posited that young children in the preoperational stage attribute animation to all things—as flawed. Huang argued that children’s tendency to label objects as “living” reflected not a worldview but the misuse of ordinary language (Huang, 1943; Huang and Lee, 1945). (2) The agent approach reinterprets the ambiguous term “living” as signifying autonomous activities, equating animacy perception with the perception of agency capable of self-propelled motion (Westfall, 2023). In this article, we also frequently employ the term “agent.” While this approach is fruitful in philosophical discussions, the notion of “agent” lacks an operational definition in cognitive neuroscience, and empirical research on sense of agency and animacy perception remains distinct, with no clear synthesis to date. (3) The integrative approach based on first-person phenomenon of animacy perception. Neither of the aforementioned approaches fully accommodates the practical demands of empirical research in cognitive neuroscience. Human perception of animacy no longer hinges on naive or scientific biology (i.e., “living” vs. “nonliving”) nor on common-sense or scientific psychology (i.e., autonomous vs. non-autonomous). Rather, neuroscience now seeks evidence of the activation of neural pathways specific to animacy in subjects’ brains, using operationalized methodologies. Of course, this is grounded in subjects’ first-person phenomenal experiences. Jozwik et al. (2022) implied five dimensions on defining animacy: being alive, looking like an animal, having mobility, possessing agency, and exhibiting unpredictability. While each dimension is somewhat reasonable, together they might provide an integrative account of animacy. We think that defining animacy, perhaps in a way analogous to the methodology of neurophenomenology, requires integrating first-person subjective reports with third-person neuroscientific empirical research. There is a one-to-one correspondence between a person’s phenomenal perception of the animate-inanimate continuum and the activation of distinct brain regions associated with these objects, such as faces, bodies, artifacts, and natural objects (Ayzenberg and Behrmann, 2024; Bracci and Op de Beeck, 2023; McMahon et al., 2023; Rosa Salva et al., 2015). For a diagrammatic representation of this part of Heider’s system reformulated based on hierarchies of animacy, see Figure 1.^1^

## 4 Concluding remarks

Heider’s theory of social cognition began to gain widespread dissemination in the 1940s through the publication of his work and the circulation of his unpublished manuscripts. However, it was not until the publication of his magnum opus, *The Psychology of Interpersonal Relations*, in 1958 that his social cognition system was fully established. Afterward, social psychologists primarily developed the attribution approach; and the paradigm shift in psychology which led to a certain disruption of the early social cognition tradition within this discipline’s internal history. While the attribution approach is related to Heider’s early causal analysis of perceptual realism concerning objects, this line of research—or rather this interpretive approach to Heider’s system—is, in our view, neither direct nor comprehensive. This is because the objects involved in event attribution, in conjunction with their intrinsic properties, have not been endowed with a constitutive scientific explanation. One central aim of this paper is to provide a constitutive analysis of general objects based on animacy perception.

Considering the relationship between Heider’s system and contemporary theories of social cognition, the former is either overlooked in discussion or roughly categorized as a precursor to theories such as folk psychology or theory theory. However, this interpretation is problematic. It is crucial to emphasize that Heider’s system has little direct historical connection to the dominant theories in contemporary philosophy of social cognition. Philosophers of cognitive science who have developed folk psychology or theory theory since the 1980s appear not to have significantly engaged with Heider’s social cognition. Beyond historical genealogies, what is more important is that Heider’s common-sense psychology is neither theory-based nor grounded in mentalistic frameworks (resorting to propositional attitudes). As Heider noted, “…the ordinary person has a great and profound understanding of himself and of other people which, though unformulated or only vaguely conceived, enables him to interact with others in more or less adaptive ways” (Heider, 1958, p. 2). Designed to serve as a foundation for scientific psychology, Heider’s common-sense psychology is nonetheless non-theoretical and unsystematic—what he described as being “unformulated or half-formulated” (p. 4). Indeed, much of its “common-sense” characteristic seems closer to intuition.^2^

Furthermore, when considering Heider’s system in relation to the contemporary “new theories” of social cognition that challenge theory theory and simulation theory—such as direct social perception and interactionism—Heider seems not to have placed significant emphasis on the directness of person perception or the importance of second-person interactivity. The historical connection between Heider and these new theories is also quite tenuous.

This article proposes a possible new way of reading to the main features of Heider’s social cognition: Heider’s system suggests a strong continuity between social perception and social structures (networks). Heider (1958) departs from the traditional social psychology concerned with how a person perceives and thinks about superindividual, social polyads or groups, focusing instead on person perception. Even when examining the perception and interaction between persons within a dyad (e.g., between “I” and “you”), Heider considers the structure of dyads (understood as one social unit) which imposes strict constraints on social cognition. The number of persons in small groups, for instance, represents an important aspect of these structural constraints. In essence, one person’s basic capacities for social cognition are continuous with the complexity of the social network in which the person is embedded; conversely, the complexity of social networks requires persons to develop sufficiently sophisticated social cognitive abilities to navigate and operate within them. A person’s balanced state cannot be achieved by considering only mentalistic dimensions—it must also resort to the complexity of the social network the person inhabits.^3^

This paper explores the relationship between animacy perception and Heider’s system, with a particular focus on the utilization of scientific evidence. Animacy perception is better understood as a more fundamental, antecedent capacity compared to mindreading or theory of mind. The capacity to comprehend specific mental contents is contingent upon the specification of an entity as a “person” (or as “minded”) rather than a “thing.” Nevertheless, the precise cognitive architecture underlying animacy perception remains contested. Contemporary discourse encompasses deliberations concerning the system’s configuration, whether singular, dual, or multiple, and the necessity of higher-order capacities, such as metacognition, for its functionality (Asif, 2022; Butterfill and Apperly, 2013; Carruthers, 2017; Christensen and Michael, 2016). While the activation of mirror neurons is limited to similar or identical actions (Dong et al., 2025); it occurs at the subpersonal rather than the personal level. The neural mechanisms associated with animacy appear to generalize across all objects (entities) encountered in the social world (Bracci and Op de Beeck, 2023; Rosa Salva et al., 2015). From a person-centered perspective, the perception of animacy primarily concerns neither the construction of subpersonal neural pathways in the brain nor the detailed decomposition of the perceived animated image. Rather, animacy perception involves the specification and recognition of the total features that make up what a “person” is—similar to how, during early social perception, person perception is primarily focused on understanding what a “person” is.^4^

## Data Availability

The original contributions presented in the study are included in the article/supplementary material, further inquiries can be directed to the corresponding authors.

## Footnotes

^1^We appreciate the reviewer’s suggestion to enhance visualization through the inclusion of Figure 1.

^2^Ratcliffe (2007) explicitly distinguishes Heider’s concept of common-sense psychology from folk psychology. Furthermore, in Heider (1958), common-sense psychology is referred to as a form of “knowledge.” However, Heider’s use of the word “knowledge” is radically different from its meaning in contemporary epistemology. According to Heider, “common-sense” does not seem to consist of propositions, as many aspects of it cannot be asserted or uttered in some sense. There are many unresolved issues surrounding the reading of Heider’s system.

^3^With respect to the study of social structures or networks, focusing solely on their formal aspects (such as the number of members) and disregarding their material aspects aligns with Georg Simmel’s formal sociology (Simmel, 2009). In a similar vein, Heider’s early theory of social cognition could be described as “formal” social cognition.

^4^We are grateful to the reviewer for suggesting a discussion in the final section on the relationship between Heider’s theory and contemporary social cognition theories. This recommendation prompted us to undertake a substantial revision of the concluding subsection of the paper.
